# Influence of personal and environmental factors on mental health in a sample of Austrian survivors of World War II with regard to PTSD: is it resilience?

**DOI:** 10.1186/1471-244X-13-47

**Published:** 2013-02-04

**Authors:** Ulrich S Tran, Tobias M Glück, Brigitte Lueger-Schuster

**Affiliations:** 1 Faculty of Psychology, University of Vienna, Liebiggasse 5, Vienna, A-1010, Austria

## Abstract

**Background:**

War-related traumata in childhood and young-adulthood may have long-lasting negative effects on mental health. The focus of recent research has shifted to examine positive adaption despite traumatic experiences, i.e. resilience. We investigated personal and environmental factors associated with resilience in a sample of elderly Austrians (N = 293) who reported traumatic experiences in early life during World War II and subsequent occupation (1945–1955).

**Methods:**

After reviewing different concepts of resilience, we analysed our data in a 3-phased approach: Following previous research approaches, we first investigated correlates of PTSD and non-PTSD. Secondly, we compared a PTSD positive sample (sub-threshold and full PTSD, n = 42) with a matched control sample regarding correlates of resilience and psychometrically assessed resilience (CD-RISC). Thirdly, we examined factors of resilience, discriminating between psychologically healthy participants who were exposed to a specific environmental stressor (having lived in the Soviet zone of occupation during 1945–1955) from those who were not.

**Results:**

A smaller number of life-time traumata (OR = 0.73) and a medium level of education (OR = 2.46) were associated with better outcome. Matched PTSD and non-PTSD participants differed in psychometrically assessed resilience mainly in aspects that were directly related to symptoms of PTSD. Psychologically healthy participants with an environmental stressor in the past were characterized by a challenge-oriented and humorous attitude towards stress.

**Conclusions:**

Our results show no clear picture of factors constituting resilience. Instead, most aspects of resilience rather appeared to be concomitants or consequences of PTSD and non-PTSD. However, special attention should be placed on a challenge-oriented and humorous attitude towards stress in future definitions of resilience.

## Background

There is evidence to suggest that children and persons of younger age are at greater risk of developing PTSD after exposure to war-related and other trauma [1]. In particular, research has found long-lasting effects of childhood and early adult trauma during World War II (WWII) on the mental health of elderly survivors studied decades after exposure, e.g., [2-8].

The emphasis of past research has been on adverse mental health outcomes following trauma but in recent years, there has been a shift towards a focus on resilience [9], the notion that survivors can manifest positive adaptations after traumatic experiences [10]. So far, resilience has been investigated in different contexts [11-15], and also among elderly persons with traumatic experiences during WWII [16-18].

### Concepts of resilience

Although resilience has received considerable attention in recent years, no strict consensus exists on how to define and operationalize it. Some authors conceive resilience as the ability to recover from extreme experiences [19], some as a reflection of general symptom improvement [15], others as the capacity to preserve a stable personal equilibrium [9]. Scholarly opinion even diverges on whether resilience may be considered as a—relatively—stable personality trait or not [10,19-21].

Resilience may incorporate different and also opposing dimensions and constructs on a trait level, like, e.g., hardiness, self-enhancement, repressive coping, or positive emotion [9] (for a ‘multiple pathways’ view on resilience; see also [22]). However, it may also depend largely on context [21]. Furthermore, resilience may also require a life-span perspective on the processes involved, as these may vary across age groups [13]. Aggravating this conceptual confusion is the sheer number of psychometric inventories of resilience currently available. Windle et al. [23] identified 15 scales that may measure each as “an entirely different experience” (p. 1). Overall, the quality of most instruments was rated at best moderate. Alternative, outcome-oriented definitions to tackle this conceptual problem, like equating “resilience = absence of PTSD symptoms” [24], may thus appear both simple and elegant. Currently, it is mostly unclear to which extent specific outcome-oriented and psychometric definitions and approaches agree and match with one another. Systematic investigation is needed to gain more insight into commonalities and differences.

### Risk and protective factors: or just correlates, concomitants, and consequences of PTSD?

Some researchers [9,25] called for the investigation of factors promoting or vitiating resilience to examine the interaction of personal and situational characteristics [22].

Research so far suffers from failing to delineate risk factors (also subsuming protective factors here) from correlates, concomitants and consequences [26] strictly enough, and from failing to disentangle different outcomes (i.e., PTSD and non-PTSD) from the components of the psychometric construct of resilience that may promote a better outcome. Any factor that shows an association with an outcome is a correlate. Risk factors precede and alter the risk of an outcome, and may be fixed or variable. They are causal if their manipulation is shown to alter the risk. If a factor does not precede the outcome, it is a concomitant or consequence.

Across studies, some putative risk factors were not consistently found to alter the risk of the outcome. Effects of personal characteristics, like, for example, age (variable marker) or sex (fixed marker) on PTSD, non-PTSD, and psychometrically assessed resilience have been equivocal and inconsistent [1,27,28]. Symptoms of depression were also reported to pose a risk for PTSD [24], when equating “resilience = absence of PTSD symptoms”. However, in the cross-sectional study of Bonanno et al. [24] precedence of depression among those who reported symptoms of PTSD was not ascertained empirically. Symptoms of depression are also characteristic of PTSD itself or may co-occur with PTSD. Hence, they may rather constitute a concomitant. This ambiguity highlights a possible drawback of the otherwise simple definition “resilience = absence of PTSD symptoms” and calls for adequate measures to guard against confounding in cross-sectional studies when using an outcome-oriented approach of research.

Environmental factors that possibly promote positive mental health despite traumatic experiences and protect against PTSD are social acknowledgement [29,30], social support [31], relationships and relationship quality [32], and stable living conditions during adolescence [27]. Among the elderly, greater social engagement, defined as visiting with friends and family, was also reported to be associated with psychometrically assessed resilience [13]. Yet, with regard to the sequence of events and the definition of a risk factor [26], aspects of social support that did not clearly precede the outcome after adversity may not be considered true protective factors. Moreover, PTSD is also associated with relationship problems [33]. As a consequence, this could effectuate lower social support in PTSD. Social support could thus constitute a concomitant of non-PTSD instead of representing a protective factor against PTSD. Research needs to guard against such artefacts.

Factors relevant for coping, like humour [34] and spirituality [12], are probably best interpreted as components of the psychometric construct of resilience [9] that promote a better outcome. If individual cognitive-behavioural characteristics were present before the experience of a traumatic event and were shown to alter the risk of the outcome, they are protective factors. Otherwise, they have to be regarded as correlates (concomitant or consequence) of non-PTSD.

### Mental health in WWII survivors

A number of studies so far have investigated factors promoting or vitiating resilience in WWII survivors. Displacement during WWII was found to contribute (causally) to psychopathology [16], while for veterans and former child soldiers positive social recognition, social support and acknowledgement, as well as a positive personal evaluation of war efforts were repeatedly reported to exert beneficial effects on posttraumatic outcome [18,35,36]. The impact of a lack of social support on posttraumatic symptoms was also documented in victims of WWII mass rapes [37]. Again, these effects of social support on a better outcome may be a result of relationship problems in PTSD (see above) or are probably more indicative of posttraumatic growth than resilience [38], when they did not clearly precede traumatic events.

A causal environmental risk factor, which has not been thoroughly investigated so far may lie in differences of traumatic load in different occupational zones post-WWII. Historical data suggest that people faced a higher risk of adversity in the Soviet occupied zones than in the Western allied (France, UK, and USA) zones in Germany and Austria [39]. Austria was incorporated into the 3^rd^ Reich in 1938 and liberated in 1945. Occupation by Western Allied and Soviet troops lasted from 1945 to 1955. In a sample of civil survivors of WWII [40], now 65+ years old, who experienced various kinds of trauma during their wartime-childhood and adolescence, over 92% reported experiences of war-related traumata or traumatic experiences with the occupational forces (termed generically WRTs in the following), and over 97% reported at least one lifetime-trauma. Prevalence of PTSD was 1.9% in this sample, but this rate increased to 14% taking into account sub-threshold PTSD. Specifically, even though not indicative of higher rates of PTSD, traumatic experiences with the occupational forces were reported more often by residents of the former Soviet occupied zone (56.5% vs. 16.7%).

### This study

Resilience has been studied in samples of elderly persons with specific war-time histories such as veterans but to our knowledge, not in more general samples of WWII survivors who experienced various kinds of trauma during their wartime-childhood and adolescence. In this study, we asked for differences and correlates between PTSD and non-PTSD individuals in such a general sample of WWII survivors. Correlates might be specific personal and situational features and characteristics like, for example, active coping, humour or social support, that may enhance the individual cognitive and behavioural capacity to cope with adverse events and to adapt to a given environment [34,41].

We contrasted and combined outcome-oriented and psychometric approaches to disentangle symptoms of PTSD from personality characteristics and cognitive-behavioural components of the psychometric construct of resilience that may promote a better outcome. This strategy allowed us to arrive at a clearer picture of risk factors, correlates, and consequences of PTSD and non-PTSD. It also enabled the investigation of the impact of environmental variables and individual coping strategies on PTSD and non-PTSD to be examined in more detail. Data previously presented [40] were used.

A 3-phased approach was used in analyzing the data. First, we examined correlates of PTSD and non-PTSD utilizing the outcome-oriented approach applied by Bonanno et al. [24], allowing thereby direct comparisons with previous results. Specifically, we examined effects of social support and acknowledgment on coping with WRTs in this phase. Moreover, we assessed whether a short and reliable psychometric measure, the 10-item Connor-Davidson Resilience Scale (10-item CD-RISC; [42]), also discriminated between the different levels of outcome according to the criteria of Bonanno et al. This allowed us to examine the extent to which the outcome-oriented and psychometric approaches overlapped.

In a second phase, correlates of PTSD and non-PTSD were re-investigated in a matched sample of PTSD cases and non-PTSD controls, matched with regard to sociodemographic characteristics and known risk factors of PTSD (e.g. number of life-time traumata). An examination of CD-RISC total and item scores in this matched sample was also undertaken to delineate more clearly positive components of resilience from negative outcomes in this psychometric instrument implied by the presence of PTSD symptoms.

In the third phase, we investigated which cognitive-behavioural characteristics were indicative of having successfully coped with an environmental risk factor in the past among those who were overall ‘resilient’ in a positive outcome-oriented sense [9]; i.e., able to preserve a stable personal equilibrium. Residents of the Soviet occupation zone post-WWII had a higher risk of adversity than residents of the Western zones [39]. Thus we compared ;CD-RISC scores of persons whose psychological health was above average at the time of the assessment (whose self-reported overall symptom severity did not exceed the 50th percentile of the population norm) from the two occupational zones (i.e., the West and Soviet zones).

## Methods

### Participants and procedure

Three-hundred-and-sixteen participants were recruited for a study on PTSD in the elderly in Austria between March and September 2010 by local announcements and through referral by institutions and residences for the elderly [40]. Participants were born before 1945 and had resided in Austria during the war and time of occupation (1945–1955). More participants were recruited from the former Soviet controlled regions. This was in accordance with population density statistics of Austria (more people live in the eastern parts of the country) [43]. Moreover, corresponding to historical reports [39], a higher traumatic load was expected there. The study was conducted according to the Austrian ethical regulations for clinical research and approved by the committee of the funding organization (Future Fund of the Republic of Austria). All participants provided informed written consent and were interviewed in their homes by expert interviewers trained in psychotraumatology and gerontology (psychology masters/PhD students).

Part of this interview (see Instruments) was a Mini Mental State Examination (MMSE; [44]). Persons with a score below 22 were excluded. The slightly lowered cut-off (22 instead of 24) was deemed more suitable for this sample where education was mostly expected to be low [45].

For this study, only the data of 293 participants, who had experienced at least one WRT, were used. Among these, MMSE scores had a mean of 27.1 (*SD* = 2.2, Range = 22–30). Twenty (6.8%) participants had a MMSE score < 24. Further sample characteristics are given in Table 1.

**Table 1:** Sample characteristics

**Sex**	**Male**	**Female**	
*N* (%)	113 (38.6)	180 (61.4)	
**Age**	*Mean* (*SD*)	Range	
Years	82.1 (6.6)	66–99	
**Education**^a^	< 10 years^b^	10–12 years^c^	> 12 years^d^
*N* (%)	144 (49.1)	109 (37.2)	39 (13.3)
**Marital status**	Single	Married	Widowed or divorced
*N* (%)	24 (8.2)	123 (42.0)	146 (49.8)
**Traumata**	War-related^e^	Other lifetime^f^	Total lifetime
*Mean* (*SD*)	1.8 (1.0)	1.8 (1.4)	3.6 (1.7)
**Zone**^a^	Western Allied	Soviet	Both
*N* (%)	117 (40.1)	170 (58.2)	5 (1.7)
**Status**^g^	Resilient	Mild-to-moderate trauma	PTSD (full or sub-threshold)
*N* (%)	170 (58.0)	81 (27.6)	42 (14.3)^h^
**With respect to status**			
**Symptoms of depression**			
*N* (%)	6 (3.5)	5 (6.2)	8 (19.0)
**Voluntary work**			
*N* (%)	49 (28.8)	18 (22.2)	4 (9.5)
**CD-RISC total score**			
*Mean* (*SD*)	31.29 (6.89)	28.59 (6.71)	25.93 (6.65)

^a^*N* = 292. Attained degree of education: ^b^ primary education or lower secondary education; ^c^ upper secondary education or vocational education and training; ^d^ university. ^e^ Includes bombing, civilian WRTs, WRTs by occupational forces, war effort, prisoner of war. ^f^ As determined with the TLEQ. ^g^ Based on Bonanno et al.’s [24] criteria. ^h^ Full PTSD was present in 6 (2.0%) participants.

### Instruments

The interview comprised the MMSE, a biographical and historical section created in cooperation with historians from the Ludwig Boltzmann Institute for War-Research in Graz, Austria, and a battery of standardized psychological instruments, see [40] for details.

The MMSE is a widely used screening instrument for dementia and cognitive impairment with high inter-rater and test-retest reliability, and high sensitivity and specificity [44]. MMSE scores range from 0 to 30. Lower educational levels were reported to impact MMSE scores [45]. Hence, a lower cutoff of < 22 (compared to < 24) was used in this study where participants had predominantly only lower levels of education.

In the biographical section of the interview, three single items (scored yes/no) assessed whether participants had received social support and acknowledgement with regard to their war-time experiences. They were used to investigate the impact of social support and acknowledgement on the successful coping specifically with WRTs. Participants were asked (1) whether they had had the opportunity to talk openly about their war-time experiences with someone, (2) whether family and/or friends were sympathetic towards their war-time experiences and (3) considered their war-time experiences as important. Questions (1)–(3) had a Cronbach’s alpha of .60 in our sample. We constructed an ad-hoc scale by adding together respective ratings, assigning 0 = *no* and 1 = *yes*. A fourth item assessed (4) whether the occupational forces had given participants a feeling of safety. We also used this item to investigate any positive contributions made by occupational troops towards their coping with WRTs.

Additionally, as indicators of social engagement and support [13], we used data from the biographical section on whether participants were currently involved in voluntary work, their marital status, and on whether they had children and were still in contact with them.

The life-time frequency of 17 different DSM-IV A-criterion traumatic experiences was assessed with the Traumatic Life Event Questionnaire (TLEQ; [46]). The TLEQ is a 19-item broad-spectrum measure of trauma exposure covering natural disasters to physical assaults and rape. It demonstrated good content validity and discriminative validity with regard to PTSD status, and good test-retest reliability [47]. For the purposes of this study, WRTs were not assessed with the TLEQ. Instead, the experience of five different categories of WRT (i.e., bombing, civilian WRTs, WRTs by occupational forces, war effort, and prisoner of war) were assessed with the biographical and historical sections of the interview. The items were designed by the historians, taking into account historical data on traumatic and other relevant events specific to that time. Formulations of items pertaining to WRTs were matched to the format of the TLEQ. For the purpose of this study, the total number of experienced WRTs was used.

The Posttraumatic Stress Disorder Checklist – Civilian Version (PCL-C; [48]) was used for the assessment of symptoms of posttraumatic stress according to DSM-IV criteria. The PCL-C asks for the occurrence and severity of 17 PTSD symptoms in the past month, scored from 1 = *not at all* to 5 = *extremely*. The PCL-C was reported to show overall good validity and reliability (see [49], and references therein). Ratings in the PCL-C were used for referencing PTSD (1+ symptoms of criterion B, 3+ symptoms of criterion C and 2+ symptoms of criterion D) and sub-threshold PTSD (1+ symptoms out of criterion B, C and D; [50]). The PCL-C was also used to place participants into the categories proposed by Bonanno et al. [24]: ‘PTSD’ (according to the above criteria but also including sub-threshold PTSD in the current study), ‘mild-to-moderate trauma’ (2+ PTSD symptoms but no full or sub-threshold PTSD), and ‘resilient’ (one PTSD symptom at most). ‘Mild-to-moderate trauma’ and ‘resilient’ may be regarded as two graduations of non-PTSD in the current context. Bonanno et al. used the National Women’s Study PTSD module to assess symptoms of PTSD but otherwise the same criteria. The only other deviance of the current study from Bonanno et al. [24] lay in including also probable sub-threshold PTSD in the ‘PTSD’ category. Research suggests that the symptom burden and distress implied by sub-threshold PTSD also affects mental health to a great extent [51,52]. Hence, sub-threshold PTSD was also considered in the current study.

A psychometric measure of resilience was obtained with the 10-item CD-RISC [42]. Items pertain to tolerance of change, coping with stress and personal problems, illness and recovery, as well as dealing with challenges, pressure, failure and painful feelings in the last month, scored from 0 = *not true at all* to 4 = *true nearly all of the time*. The 10-item CD-RISC was shown to be a reliable, valid, and one-dimensional measure and is thought to capture the core features of resilience, defined as the ability to cope with stress [23]. In the comprehensive review of Windle et al. [23] the 10-item CD-RISC was rated as one of the better short instruments currently available.

Current general distress and depressive symptoms were assessed with the German version of the Brief Symptom Inventory (BSI; [53]). The Global Severity Index (GSI; mean of all 53 items) was used to identify participants whose psychological health was above average at the time of the assessment (T scores < 51, based on the adult norm). Being able to preserve a stable personal equilibrium [9] applies to a healthy and normal functioning as a whole and hence implies a lack of symptoms across the entire spectrum. The GSI is a highly reliable measure of global symptom distress [53]. Thus, it also appeared to be suited as an indirect indicator of overall healthy functioning. Symptoms of depression as assessed with the BSI depression subscale (6 items) were regarded as clinically relevant in participants with T scores > 62.

### Analyses

In the first phase of analysis, multinomial logistic regression was used, in analogy to [24]. The current status of participants (resilient/mild-to-moderate trauma/PTSD) was used as outcome and ‘PTSD’ was set as the reference category. Current status was regressed on participant sex, age, education, number of life-time traumata (combining WRTs and non-WRTs), and current depression, comparable to [24]. Furthermore, voluntary work and marital status served as indicators of social engagement and support. Current residence (nursing home/own home) was included in our model to control for further possible confounding (107 [36.5%] participants resided in nursing homes). The association of social support and acknowledgement with regard to WRTs with status was tested by including the ad-hoc scale of questions (1)–(3). Respective scores ranged from 0 to 3 with *M* = 1.81 (*SD* = 1.08). Question (4) on positive contributions of occupational troops (approved by 95 [32.4%] participants, 15 missings) was added to the model as a categorical regressor. Differences in CD-RISC total scores between the three status groups were investigated with analysis of covariance, controlling for age and total number of life-time traumata.

For the second phase of analysis, we created a matched case–control sample, wherein the 42 participants with full or sub-threshold PTSD were matched with 42 non-PTSD controls of our sample (matching criteria: sex, age, education, residence during WWII [city/countryside], number of life-time traumata, and MMSE score). Of the matching criteria, sex, age, education, and number of life-time traumata were known risk factors of PTSD [1]. We (re-)analysed in this matched case–control sample all predictors that were examined in the first part of our analysis that were not matching criteria (i.e., symptoms of depression, voluntary work, marital status, current residence in nursing home), but also effects of zone of occupation, whether participants had children, and differences in CD-RISC total and single item scores using *t* tests, Fisher’s exact tests, and likelihood ratio tests.

For the third part, we selected participants whose psychological health was at the time of assessment above-average (GSI T score < 51). Residents of the Soviet occupation zone post-WWII had a higher risk of adversity than residents of the Western zones [39]. CD-RISC item and total scores were compared between participants who had resided in the Soviet occupied zone (cases) and those who had resided in the Western Allied zone (controls) with *t* tests. Significance was set at *p* < .05 for all analyses.

## Results

### Correlates of resilience in the total sample

The multinomial regression model had a significant fit on the data (*χ*^2^(24) = 53.22, *p* < .001; *N* = 277 because of partially missing data). A medium level of education, a lower number of lifetime traumata, the absence of symptoms of depression, and involvement in voluntary work were associated with non-PTSD (‘resilient’) compared to PTSD (see Table 2). In contrast, mild-to-moderate trauma was associated with a lower probability of being married and a higher probability of being involved in voluntary work compared to PTSD. We also checked on differences between the ‘resilient’ and ‘mild-to-moderate trauma’ groups by setting the latter as reference category in the regression model (reporting only significant results here, *p* < .05): non-PTSD (‘resilient’) was specifically associated with a smaller number of life-time traumata (OR = 0.81, 95% CI = [0.68, 0.98]) compared to mild-to-moderate trauma. Age, sex, residing in a nursing home, social support and acknowledgement on WRTs, and positive contributions of the occupational forces on the coping with WRTs showed no significant association with any status group.

**Table 2:** Multinomial logistic regression predicting outcome (reference category = PTSD)

		**Resilient vs. PTSD**^**a**^	**Mild-to-moderate trauma vs. PTSD**^**a**^
Age		0.95 [0.94, 1.07]	1.03 [0.96, 1.10]
Female Sex		0.58 [0.23, 1.46]	0.72 [0.26, 1.99]
Education (compared to < 10 years)	10–12 years	**2.46 [1.01, 6.05]**	2.59 [0.98, 6.82]
	> 12 years	1.57 [0.43, 5.72]	0.93 [0.20, 4.25]
Marital status (compared to	Married	0.59 [0.22, 1.59]	**0.26 [0.87, 0.80]**
widowed or divorced)	Single	0.84 [0.14, 5.09]	1.74 [0.30, 10.24]
Living at own home (compared to nursing home)		1.02 [0.41, 2.49]	0.91 [0.35, 2.37]
Number of lifetime traumata		**0.73 [0.59, 0.91]**	0.90 [0.71, 1.14]
Symptoms of depression		**0.21 [0.06, 0.71]**	0.29 [0.08, 1.04]
Engaged in voluntary work		**4.86 [1.42, 16.70]**	**4.34 [1.12, 16.79]**
Social support on WRTs		0.91 [0.64, 1.29]	1.00 [0.68, 1.47]
Positive contribution of occupational forces		0.82 [0.37, 1.80]	0.49 [0.21, 1.19]

Odds ratios (ORs) and 95% confidence intervals. Significant (*p* < .05) ORs are printed boldface. ^a^ Full and sub-threshold PTSD.

Current status had a significant impact on CD-RISC total scores (see Table 2) with a medium effect size, *F* (2, 283) = 12.04, *p* < .001, η^2^ = .078. Bonferroni-corrected, non-PTSD (‘resilient’) participants differed significantly from mild-to-moderate traumatized participants (*p* = .011) and from participants with probable PTSD (*p* < .001). Mild-to-moderate traumatized participants and participants with probable PTSD did not differ (*p* = .105). The covariates had no significant impact (age: *F* (1, 283) = 0.03, *p* = .874; number of life-time traumata: *F* (1, 283) = 1.20, *p* = .274).

### Comparison of PTSD cases with matched controls

Cases and controls matched perfectly with regard to sex, educational level, and residence during WWII. They were comparable regarding all other matching criteria (age: *M* = 82.05 vs. 81.98, *t*(82) = 0.06, *p* = .952; total number of life-time traumata: *M* = 4.24 vs. 4.07, *t*(82) = 0.43, *p* = .668; MMSE score: *M* = 26.64 vs. 27.21, *t*(82) = −1.28, *p* = .205). 13 of the controls were classified as mild-to-moderate trauma. This was inevitable, as a larger number of traumata (which served as a matching criterion) raised the probability of showing symptoms of PTSD (see above).

Cases and controls differed in none of the examined variables that were no matching criteria (marital status: likelihood ratio = 3.27, *df* = 2, exact *p* = .157; symptoms of depression: OR = 3.06, 95% CI = [0.75, 12.46], *p* = .194; current residence in nursing home: OR = 1.22, 95% CI = [0.51, 2.96], *p* = .822; voluntary work: OR = 0.39, 95% CI = [0.11, 1.37], *p* = .227). They also did not differ with regard to zone of occupation (Soviet zone: OR = 0.74, 95% CI = [0.31, 1.78], *p* = .658). However, cases more often had children (*n* = 38 vs. 29, OR = 4.26, 95% CI = [1.26, 14.43], *p* = .028). The mean number of children among those with children did not differ between cases and controls (*M* = 2.11 vs. 1.90; *t*(65) = 0.70, *p* = .489). All cases and 26 of the controls reported that they also had regular contact with their children.

Total CD-RISC scores differed between cases and controls by a large amount (see Table 3). With regard to items, differences were significant in Items 6, 10, 7, 8, 2 and 5 (in descending order with regard to effect size) and there at least of medium size. No significant differences were found for Items 1, 3, 4, and 9.

**Table 3:** Differences in CD-RISC item and total scores in the matched case–control sample

**Item**	**Cases**	**Controls**	***t***	***p***	***d***
1. Able to adapt to change	3.23 (1.10)	3.36 (1.14)	−0.53^a^	.595	−0.12
2. Can deal with whatever comes	2.66 (1.10)	3.21 (0.93)	−2.48^b^	.015	−0.54
3. Tries to see humorous side of problems	2.05 (1.30)	2.45 (1.44)	−1.34^b^	.184	−0.29
4. Coping with stress can strengthen me	1.27 (1.38)	1.81 (1.57)	−1.67^b^	.099	−0.37
5. Tend to bounce back after illness or hardship	3.15 (0.91)	3.60 (0.73)	−2.43^a^	.018	−0.54
6. Can achieve goals despite obstacles	2.56 (1.42)	3.40 (0.91)	−3.24^b^	.002	−0.71
7. Can stay focused under pressure	2.58 (1.28)	3.24 (1.06)	−2.57^a^	.012	−0.57
8. Not easily discouraged by failure	2.68 (1.25)	3.31 (1.05)	−2.50^a^	.015	−0.55
9. Thinks of self as strong person	2.95 (1.32)	3.33 (0.85)	−1.57^b^	.120	−0.35
10. Can handle unpleasant feelings	2.63 (1.22)	3.38 (1.06)	−2.98^b^	.004	−0.65
Total score	25.93 (6.65)	31.10 (6.12)	−3.65^a^	< .001	−0.81

^a^*df* = 80, ^b^*df* = 81.

### Successful coping with an environmental risk factor in the past

In our sample we identified 173 participants whose psychological health was above average (GSI T score < 51) at the time of the assessment, 94 (54.3%) of whom had resided in the Soviet zone (cases) and 79 (45.7%) in the Western Allied zone (controls). Cases differed from the controls in CD-RISC total scores by *d* = 0.39 (*M* = 32.86 vs. 30.49, *t*(170) = −2.57, *p* = .011); i.e., the cases had higher scores. In single item analyses, this difference could be traced to Items 3 (‘humour’; *M* = 2.98 vs. 2.29, *t*(171) = 3.51, *p* < .001, *d* = 0.54) and 4 (‘stress strengthens’; *M* = 2.23 vs. 1.54, *t*(171) = 3.03, *p* = .003, *d* = 0.46). The cases and controls did not differ in any of the other items (*p*s ≥ .088). Ratings in Items 3 and 4 were in both the cases and controls slightly positively correlated, but this was significant only with regard to the cases (Spearman rho = .21 and .18, *p*s = .040 and .120). Dichotomizing items at a rating of 2 (*sometimes true*), the cases had a more than eightfold chance to rate one item at least *sometimes true*, provided they had also rated the other item at least *sometimes true* (OR = 8.07, 95% CI = [2.07, 31.47], *p* = .002). No such association was observable among controls (OR = 1.98, 95% CI = [0.77, 5.14], *p* = .235).

## Discussion

Our study shows that outcome oriented and psychometric research approaches on resilience converged to some extent. Yet, each was deficient in its own way. By using different research vistas and diligent control for confounding, we were able to avoid bias and to identify more clearly predictors and correlates of positive mental health despite trauma. Our findings corroborate some previous results but also expand these findings with regard to the elderly who had traumatic experiences during their childhood and adolescence.

With regard to the outcome oriented approach, comparing persons with mild-to-moderate trauma or PTSD with non-PTSD (‘resilient’) persons, a higher number of life-time traumata and current depressive symptoms were found to be associated with current symptoms of posttraumatic stress, corroborating previous results [24,54]. While the number of previous traumata may be reliably regarded as a variable risk factor, analyses with our matched case–control sample suggest that depression is most likely no true risk factor but rather a concomitant of PTSD and its symptoms. Evidence substantiating this conclusion in the elderly was recently reported by Chaudieu et al. [55]. Symptoms of depression may thus indicate current posttraumatic stress rather than pose a risk factor of PTSD in the elderly. This needs consideration in clinical treatment.

A medium level of education, compared to a low level, also appeared beneficiary for non-PTSD in our study. Previous studies reported conflicting evidence on this issue [11,24]. Judging from our data, a high level of education does not impede adaptation to trauma [24]. It rather seemed to have no specific beneficial effect compared to a lower level of education. The effects of education need to be investigated more specifically in future research.

Sex did not emerge as a risk factor in our study, corroborating findings by Spitzer et al. [7] in a community sample of elderly Germans. However, full and sub-threshold PTSD was more frequent among married persons in our study, compared to persons with some symptoms of PTSD but no probable diagnosis. Matching with regard to marital status and a number of other sociodemographic characteristics, persons with full or sub-threshold PTSD were also more likely to have children. These findings are somewhat at odds with previous results on higher levels of perceived social support [18,24,27] and greater social engagement [13] in resilient persons. Yet, our findings may reflect an aspect of help-seeking behaviour in persons with PTSD. Recent research suggests that spouses’ emotion-focused coping strategies may have a beneficial impact on victims’ PTSD symptoms [56]. Stronger familial ties and an increased likelihood to rear children may be a consequence of this kind of help-seeking behaviour. Marital status and a higher likelihood to rear children may in this respect be regarded as consequences of PTSD in the elderly. More research is, however, needed on this topic.

Voluntary work in old age was also associated with a lower probability of full or sub-threshold PTSD. In absence of longitudinal data, we suggest that this may be inversely interpreted as a consequence of the debilitating symptoms of PTSD: persons with PTSD may be less able — because of their symptomatology — to involve themselves in such activities [57]. Volunteerism may thus be understood as an indicator (i.e., a concomitant or consequence) of non-PTSD but not as a protective factor against PTSD.

In contrast to other reports on positive posttraumatic outcome [30,35], PTSD and non-PTSD were not characterized by differences in social acknowledgement or of having had the opportunity to talk openly about war-time experiences with someone. These conflicting results may be due to sampling differences: Forstmeier et al. [35] investigated former WWII child soldiers, whereas Maercker and Müller [30] studied survivors of political imprisonment in former Eastern Germany and recently traumatized crime victims. These samples may have been representative of persons who had some ‘special’ or uncommon traumatic experience. Most civilian WWII child-survivors are not recognized as having a special or in some way outstanding history to tell. Consequently, social acknowledgement and the seeking of such may be generally lower in the cohorts of civilian WWII child-survivors. While social acknowledgement could have been beneficial to them, they might not have had the chance to acquire it.

With regard to the psychometric approach, the 10-item CD-RISC was found to discriminate reliably between PTSD and non-PTSD persons in our study. In the matched case–control sample, differences were accordingly also greatest in items that were related to PTSD symptoms of clusters B and D (Items 6, 7, and 10). Thus, resilience as measured with the CD-RISC evidently mirrored to a large extent only PTSD symptom severity, calling the utility of the CD-RISC somewhat into question [54]. Moreover, being able to adapt to changes (Item 1), considered an essential indicator of psychometrically defined resilience [15], did not discriminate between matched PTSD and non-PTSD persons. Yet, among those whose psychological health was above average, seeing the humorous side of problems (Item 3) and maintaining the impression that coping with stress can be strengthening (Item 4) were found to be indicative of having dealt successfully with an environmental risk factor in the past. This study thus corroborated that humour is an important component of resilience and coping [34,58]. Yet, our study also shows that it is complemented by a challenge-oriented attitude towards life. We suggest that these two inter-related cognitive-behavioural characteristics should be regarded as protective factors. Fostering these two factors could thus be important for prevention programmes that seek to boost resistance against posttraumatic stress and PTSD. Experimental and longitudinal research is needed here.

With regard to the initially posed question (‘Is it resilience?’), our study provides no definite answer. We obtained evidence of a risk and a protective factor (number of life-time traumata, medium education), and of a number of likely correlates and consequences of PTSD and non-PTSD in the elderly (symptoms of depression, voluntary work, marital status, likelihood to rear children). From this perspective, only fewer traumata and a medium level of education appeared to promote better mental health and resilience (i.e., showing less likely symptoms of PTSD), replicating previous results [24]. Humour and a challenge-oriented attitude towards life were found to be important aspects in coping successfully with an environmental risk factor in the past. However, these characteristics did not discriminate PTSD from non-PTSD. Thus, our study’s main contribution may lie in pointing out that the question ‘*What* is resilience?’ needs reformulation. Studies need to examine in more detail which specific factors contribute to good mental health in which specific way. Likewise, studies need to differentiate more systematically between different levels of outcome (e.g., PTSD and non-PTSD), types of correlates (i.e., risk factors, concomitants and consequences), and cognitive-behavioural characteristics of psychometric definitions and operationalizations of resilience that may promote a better outcome. In conclusion, our results underline that the psychometric assessment of resilience needs improvement and should be based on a stringent definition of resilience that avoids too large an overlap with the symptomatology of PTSD. Such an instrument should incorporate — in a ‘multiple pathways’ approach — different and various components that are thought to bring resilience about or for which ample evidence already exists (i.e., humour).

Limitations of our study pertain to its cross-sectional character, which precludes direct inference on causality, problems of reporting bias given the old age of the participants and the large time spans covered, and only limited control over confounding variables that may have introduced further bias, like sampling. In the absence of normative data with regard to the base population of war-exposed Austrians, it is unclear whether our sample was truly representative. The use of ad-hoc scales and single items with regard to social support and acknowledgement on WRTs may have biased results. Associations with the Big Five personality traits and specific coping styles [32,59], which may help in mapping out the terrain of resilience as a personality trait [22], were also not considered in this study.

## Conclusions

Our results show no clear picture of factors constituting resilience. Rather, most aspects of resilience appeared rather to be concomitants or consequences of PTSD and non-PTSD. Clearer definitions of resilience and longitudinal studies are needed in future research. Special attention should be laid on a challenge-oriented and humorous attitude towards stress in order to further knowledge on resilience and on risk or protective factors that contribute to its effects.

## Competing interests

The authors declare that they have no competing interests.

## Authors' contributions

UST and TMG contributed equally to this manuscript. UST and TMG wrote the paper, planned and conducted the statistical analysis, BLS designed and supervised the project and contributed to writing and revising the paper. All authors read and approved the final manuscript.

## Pre-publication history

The pre-publication history for this paper can be accessed here:

http://www.biomedcentral.com/1471-244X/13/47/prepub
